# Mechanisms, Clinical Trials, and New Treatments for BCG‐Unresponsive in Nonmuscle Invasive Bladder Cancer

**DOI:** 10.1002/cam4.71243

**Published:** 2025-09-15

**Authors:** Xiaotong Huang, Xuan Wang, Zihe He, Yishu Huang, Bing Hu, Weiying Chen, Haifang Du

**Affiliations:** ^1^ Second Affiliated Hospital (Guangdong Provincial Hospital of Chinese Medicine) Guangzhou University of Chinese Medicine Guangzhou China; ^2^ Zhongshan Institute for Drug Discovery, Shanghai Institute of Materia Medica, Chinese Academy of Sciences Zhongshan China; ^3^ Xi'an Jiaotong‐Liverpool University Suzhou China; ^4^ Guangdong Pharmaceutical University Guangzhou China; ^5^ Guangdong Provincial Key Laboratory of Chinese Medicine for Prevention and Treatment of Refractory Chronic Diseases Guangzhou China; ^6^ Guangdong Provincial Key Laboratory of Clinical Research on Traditional Chinese Medicine Syndrome Guangzhou China; ^7^ Hunan Yueyang Maternal & Child Health‐Care Hospital Yueyang China

**Keywords:** BCG treatment, BCG vaccine replacement therapy, BCG‐unresponsive treatment, bladder cancer, gene therapy, immune checkpoint inhibitors, photodynamic therapy, targeted therapy

## Abstract

**Background:**

Bacillus Calmette–Guérin (BCG) is the standard adjuvant therapy for high‐risk non‐muscle invasive bladder cancer (NMIBC), yet treatment failure occurs in 30% to 40% of patients. Those with BCG‐unresponsive disease face a high risk of progression and represent a critical unmet need in urologic oncology. This review summarizes the mechanisms of BCG failure and evaluates emerging therapies for BCG‐unresponsive NMIBC.

**Methods:**

We conducted a comprehensive literature review of clinical trials and preclinical studies through August 2025, focusing on therapeutic strategies for BCG‐unresponsive NMIBC. Mechanisms of BCG resistance, regulatory definitions, and results from recent Phase II/III trials were analyzed.

**Results:**

Multiple novel therapies have demonstrated efficacy in BCG‐unresponsive patients. Immune checkpoint inhibitors (e.g., pembrolizumab) achieved complete response (CR) rates of 41% in carcinoma in situ (CIS) patients. Gene therapies such as nadofaragene firadenovec and CG0070 induced CR rates of 51% and 75%, respectively. Device‐assisted therapies including hyperthermic intravesical chemotherapy (HIVEC) showed 24‐month recurrence‐free survival of 57.4%. The IL‐15 superagonist Anktiva (nogapendekin alfa inbakicept), recently FDA‐approved, achieved a 71% CR rate with a median duration of 26.6 months when combined with BCG.

**Conclusions:**

The treatment landscape for BCG‐unresponsive NMIBC is rapidly evolving, with immune checkpoint inhibitors, gene therapies, targeted agents, and advanced drug delivery systems showing promising efficacy. These innovations provide bladder‐preserving options for patients ineligible for radical cystectomy. Future directions include biomarker‐driven therapy selection, combination regimens, and optimized intravesical delivery platforms to improve long‐term outcomes.

## 1 Introduction

Ranked as the ninth most prevalent cancer globally, bladder cancer significantly affects patients' quality of life, incurs substantial treatment costs, and contributes to mortality [1]. Bladder cancer is the 10th most prevalent malignancy globally, with around 573,000 new cases each year, and it ranks 13th in mortality according to 2024 global cancer statistics [2]. In high‐risk non‐muscle invasive (NMIBC) patients, bacillus Calmette–Guérin (BCG) failure occurs in 40% of cases, leading to a threefold higher risk of progression to muscle invasive (MIBC) within 5 years [3]. Bladder cancer is classified into two types: NMIBC and MIBC, with NMIBC comprising 75% of cases. NMIBC is characterized by a significant recurrence rate, ranging from 31% to 78% over 5 years, contributing to a substantial disease burden [4, 5]. In NMIBC, low‐grade Ta uroepithelial carcinoma often recurs but rarely progresses. The five‐year progression rate for carcinoma in situ is 50%, and the probability that a patient with untreated carcinoma in situ will progress to MIBC within 5 years is between 40% and 80% [6, 7].

For NMIBC, the standard initial treatment is transurethral resection of bladder tumor (TURBT), and BCG is the main adjuvant therapy for cases at high risk of progression [8]. BCG not only inhibits cell proliferation and leads to the death of bladder cancer cells through direct cytotoxicity, but also inhibits tumor growth by activating the immune microenvironment of the bladder and inducing polarization of immune cells toward the antitumor M1‐type, thus effectively reducing the recurrence and progression rates [9].

However, BCG treatment still has limitations. In terms of vaccine supply, BCG vaccine is in short supply due to production difficulties and increased demand, which affects the accessibility of treatment. BCG vaccination is not fully effective, with 30% to 40% of patients either not responding or experiencing relapse posttreatment, highlighting the urgent need for alternative therapies for BCG vaccine failure [10]. For adverse effects, BCG may cause local or systemic side effects, which are poorly tolerated by some patients.

BCG‐unresponsive is a regulatory‐defined subset referring to patients who experience Ta/T1 recurrence within 6 months or carcinoma in situ (CIS) recurrence/persistence within 12 months of adequate BCG therapy (including induction and maintenance), with little to no expected benefit from additional BCG instillations [11, 12]. This classification facilitates standardized inclusion criteria for clinical trials and guides appropriate therapy.

There are many emerging therapies being developed to ameliorate the BCG shortage and BCG‐unresponsive [13, 14, 15]. Immune checkpoint inhibitors targeting PD‐1/PD‐L1 effectively sustain effector T‐cell function, crucial for anticancer therapy [16]. Gene therapy, including adenosine, CG0070, and BC‐819, is in advanced clinical development stages. Photodynamic therapy employs light energy to destroy malignant urothelial cells. Targeted therapies, including IDO and EpCAM, present promising targets for bladder cancer treatment and are currently under evaluation in clinical trials for BCG‐unresponsive cases.

Based on recent developments in clinical trials, this article focuses on alternative therapies to BCG therapy and the latest research findings by describing the history of BCG, its mechanism of action, the definition of BCG‐naïve patients, and therapeutic strategies, and discussing the potential of various emerging therapies.

## 2 Classification and Assessment of Bladder Cancer

The bladder is a muscular organ that temporarily holds up to 500 mL of urine. The bladder wall is composed of three layers: the inner urothelium, which acts as a barrier in direct contact with urine; the middle lamina propria, a connective tissue layer offering structural support; and the outer muscularis propria, a thick muscle layer responsible for urine expulsion.

Bladder cancer refers to a tumor that originates in the bladder lining. Nearly 75% of individuals who receive a new diagnosis of bladder cancer have NMIBC, also known as superficial bladder cancer. The urothelium, the mucosal layer of the bladder wall, is the primary site of NMIBC. NMIBC is a prevalent and diverse condition characterized by frequent recurrences [17]. The cancer is located in the tissue lining the bladder's inner surface, without affecting the bladder muscle. It is a subtype of bladder carcinoma, commonly referred to as “superficial” bladder cancer. In contrast, MIBC is characterized by the presence of cancer within the bladder's muscular wall [18, 19].

The staging of bladder cancer, from NMIBC, is highly categorical to MIBC. Ta, CIS, and T1 are staging terms for NMIBC, while T2, T3, and T4 refer to MIBC. Staging evaluates the extent of cancer spread within the bladder and its potential metastasis to lymph nodes or other body parts [20]. In the superficial cancer category, around 70% are Ta lesions, 10% are CIS, and 20% are T1 lesions [21]. Ta manifests as finger‐like papillary tumors, affecting only the inner bladder layer adjacent to the urine [22]. CIS refers to cells located in the innermost layer of the bladder lining, potentially presenting as flat red patches on the mucosa or as hidden lesions not easily detected during standard cystoscopy. T1 indicates that the cancer has extended from the bladder lining into the adjacent connective tissue, often appearing nodular or papillary. Furthermore, stages T2, T3, and T4 indicate MIBC, where the cancer has penetrated the bladder muscle or extended into adjacent tissues: pelvic wall, prostate, uterus, vagina [23].

Recent studies have explored the potential of using mRNA expression levels of specific markers to aid in the staging and grading of NMIBC. Notably, the expression of ESR1, ERBB2, and Ki67 has been investigated for their correlation with tumor stage and grade. These markers, originally established in breast cancer research, have shown promising results in bladder cancer as well. For instance, higher mRNA expression levels of MKI67, ERBB2, and ESR1 have been positively correlated with higher tumor stages and grades, suggesting their potential utility in clinical practice [24].

In a study of 381 NMIBC cases, significant correlations were identified between marker expression and both the pathological stage and grade of tumors. Specifically, MKI67 expression showed a strong correlation with tumor grade, while ESR1 and ERBB2 also demonstrated significant differences between various grades and stages. These findings indicate that molecular grading using these markers could complement traditional pathological methods, potentially reducing variability and enhancing the accuracy of NMIBC assessments [24].

## 3 Risk Assessment for NMIBC and Treatment Recommendations

Most individuals with NMIBC are typically treated with transurethral resection of the bladder tumor (TURBT) [25]. This procedure aims to completely remove all visible lesions and obtain specimens for accurate pathological diagnosis and staging, which is crucial for subsequent risk assessment and treatment planning. TURBT is commonly followed by intravesical chemotherapy, with a single immediate postoperative instillation administered within 24 h being a standard approach for many patients. Intravesical therapy involves delivering the drug directly into the bladder via a soft catheter, effectively preventing post‐TURBT tumor cell implantation, eliminating residual disease, reducing tumor recurrence, and delaying or mitigating tumor progression [26, 27].

An effectively executed TURBT provides essential histopathologic data for staging and grading bladder tumors, which is vital for prognostication [25]. Further analysis of the samples can reveal details such as tumor subtype, lymphovascular invasion, and presence of carcinoma in situ (CIS), all of which influence risk stratification. After grading and staging, bladder cancer is classified into risk groups based on the likelihood of recurrence and progression. According to the 2025 European Association of Urology (EAU) Guidelines, there are four risk groups: low, intermediate, high, and very high (Table 1). This stratification considers factors such as tumor stage, grade, size, multifocality, presence of CIS, and patient age [29, 30].

**TABLE 1:** 2025 EAU guideline recommendations for NMIBC risk stratification and treatment [28].

Risk stratification	Definition	Treatment recommendations
Low Risk	– Primary, solitary Ta LG/G1 tumor, < 3 cm, without CIS, in patients ≤ 70 years – Primary Ta LG/G1 tumor without CIS and ≤ 1 additional clinical risk factor (age > 70, multifocality, tumor size > 3 cm)	– Immediate single intravesical chemotherapy instillation post‐TURB (e.g., mitomycin C) – No adjuvant chemotherapy or BCG required – Follow‐up: Cystoscopy at 3 months, then annually for 5 years
Intermediate Risk	– Patients without CIS not classified as low, high, or very high risk – Includes Ta LG/G2, T1 LG, or HG/G2 tumors with partial clinical risk factors	– Immediate single intravesical chemotherapy post‐TURB ± additional adjuvant chemotherapy (up to 1 year) – Alternative: 1‐year BCG induction + maintenance (3 weekly instillations at 3, 6, and 12 months) – Follow‐up: Cystoscopy at 3 months, every 6 months for 2 years, then annually for 10 years
High Risk	– All T1 HG/G3 tumors (without CIS) – All CIS patients (unless classified as very high risk) – Multiple high‐risk features (multifocality, recurrence, large tumor size)	– **First‐line:** 1–3 years of BCG maintenance therapy (induction +3 weekly instillations at 3, 6, 12, 18, 24, 30, and 36 months) – **Alternative:** Radical cystectomy (preferred for young/fit patients) – Follow‐up: Cystoscopy and cytology every 3 months for 2 years, then every 6 months up to 5 years, lifelong monitoring
Very High Risk	– T1 HG/G3 with CIS or ≥ 3 clinical risk factors – Presence of LVI or variant histologies (e.g., micropapillary carcinoma) – CIS in prostatic urethra or upper urinary tract	– **Strong recommendation:** Radical cystectomy – **Alternative** (for inoperable/refractory cases): 1–3 years of BCG maintenance – Follow‐up: Cystoscopy and cytology every 3 months for 2 years, then every 6 months up to 5 years, lifelong monitoring
BCG Failure Subcategories	– **BCG‐unresponsive:** Ta/T1 HG recurrence within 6 months or CIS recurrence within 12 months post‐BCG – **BCG‐relapsing:** Late recurrence after adequate BCG therapy	– **Standard:** Radical cystectomy – **Bladder preservation** (for inoperable cases): – Novel immunotherapy (e.g., PD‐1 inhibitors: Pembrolizumab) – Sequential intravesical chemotherapy (gemcitabine + docetaxel) – Device‐assisted therapies (e.g., hyperthermic MMC) B2:C6‐ Clinical trial enrollment

*Note:* EAU versus AUA divergence: EAU retains HG Ta ≤ 3 cm as high risk, whereas AUA downgrades to intermediate risk.

Abbreviations: BCG, bacillus Calmette‐Guérin; CIS, carcinoma in situ; HG, high grade; LG, low grade; LVI, lymphovascular invasion; MMC, Mitomycin C; PD‐1, programmed cell death Protein 1; TURB, transurethral resection of the bladder.

Risk stratification guides the selection of appropriate adjuvant therapy following initial TURBT. Tools like the European Organization for Research and Treatment of Cancer (EORTC) scoring model or the 2021 EAU NMIBC scoring model help estimate recurrence and progression probabilities [31]. Low‐risk tumors (e.g., primary, single Ta low‐grade tumors < 3 cm without CIS) often require only a single immediate postoperative chemotherapy instillation [32]. Intermediate‐risk tumors may be managed with additional intravesical chemotherapy or BCG immunotherapy. High‐risk and very high‐risk tumors (e.g., T1 high‐grade, CIS, or tumors with adverse subtypes/lymphovascular invasion) typically require BCG induction and maintenance therapy for 1 to 3 years, with very high‐risk cases having a significantly higher progression rate (up to 53% at 10 years) necessitating closer monitoring and potential consideration of radical cystectomy in selected cases. A second TURBT is recommended in specific situations, including incomplete initial resection, absence of detrusor muscle in the specimen (except for Ta low‐grade tumors), and all T1 tumors. This procedure, performed within 2 to 6 weeks of the initial resection, helps detect residual disease, improve staging accuracy, and enhance long‐term outcomes [12].

Intravesical BCG remains the cornerstone of adjuvant treatment for high‐risk NMIBC, with proven efficacy in reducing recurrence and progression. However, BCG shortage may necessitate alternative strategies, and combination therapies or novel agents are being explored for BCG‐unresponsive disease. The management of NMIBC thus requires a personalized approach, integrating clinical and pathological factors to optimize outcomes while minimizing treatment‐related morbidity.

## 4 History of BCG and Its Applications

*Mycobacterium bovis* ( *M. bovis* ) BCG serves two primary therapeutic purposes: as a vaccine to prevent tuberculosis and as a therapy for NMIBC patients. Its story starts from the early 20th century. In 1908, bacteriologist Albert Calmette and veterinarian Camille Guerin from the Pasteur Institute in France initiated an experiment to create a vaccine by attenuating the virulence of the bovine strain *M. bovis* [33, 34]. After 13 years of development, a live attenuated vaccine was introduced in 1921, derived from 230 serial subcultures of the pathogen *M. bovis* on glycerinated bile potato medium [34]. The BCG strain, now recognized for its reduced virulence in calves and guinea pigs, provides protection against virulent challenges and induces tuberculin sensitivity [35, 36]. Subsequently, BCG started being produced on a large scale for preventing tuberculosis in humans, and it is still the only vaccine available for this disease. BCG is the most commonly used vaccine worldwide, with more than 4 billion doses given.

In 1929, autopsy studies suggested potential antitumor effects of tuberculosis, observing a reduced incidence of cancer in affected patients. The potential application of BCG in cancer therapy was initiated by Old et al. In 1959, studies revealed that BCG‐infected mice exhibited enhanced resistance to transplantable tumors [37, 38]. BCG was initially employed as a cancer treatment in 1969 following promising outcomes reported by Mathe et al. in its use as adjuvant therapy for acute lymphoblastic leukemia. Despite several studies suggesting the potential efficacy of the new BCG vaccine in treating various cancers, this has not been confirmed. It was not until 1976 that Morales et al. firstly reported the successful use of BCG in the treatment of bladder cancer [39]. The initial success of BCG in treating bladder cancer led to its widespread adoption for this purpose. In 1990, the FDA approved BCG as the standard treatment for NMIBC. Since then, BCG has been gradually promoted for bladder cancer treatment.

Upon establishing BCG for NMIBC treatment, manufacturers adjusted the vial concentration and formulation for bladder delivery, equating one bladder cancer dose to over 4000 vaccination doses. Due to the high incidence of adverse effects from BCG treatment, ranging from mild adverse reactions to severe infections, ongoing optimization of both the induction and maintenance courses, as well as the selection of targeted patients, is being pursued through extensive trial and error.

As research progress, a large body of clinical data has demonstrated the significant efficacy of BCG bladder perfusion in preventing recurrent NMIBC after TURBT, with complete remission rates of up to 70% [40]. The standard approach for treating high‐risk NMIBC patients to prevent disease recurrence and progression is BCG [40]. After TURBT, patients receive a six‐week induction therapy with weekly intravesical instillations containing hundreds of millions of bacilli. Upon favorable therapeutic response, a maintenance regimen comprising six weekly instillations, repeated every 3 months for 1–3 years, is employed to maximize efficacy in preventing recurrence [10].

## 5 Mechanisms of BCG in Bladder Cancer Treatment

### 5.1 Direct Cytotoxic Effects

BCG exerts direct cytotoxic effects in bladder cancer treatment. Studies reveal that the attachment of BCG to bladder cancer cells through fibronectin is crucial for its treatment process [41]. Following attachment, BCG bacteria internalize into tumor cells and release *mycobacterium tuberculosis* antigens, such as MPT64 and Ag85 [42]. These antigens not only exhibit immunogenicity but also directly induce tumor cell apoptosis, thereby suppressing tumor growth. BCG therapy also leads to higher levels of antimicrobial peptides such as beta‐defensin‐2 and beta‐defensin‐3. These peptides boost innate immunity by directly eradicating pathogens and attracting immune cells to the tumor site via inflammatory signaling. For example, clinical trials have demonstrated that BCG‐treated patients exhibit significantly elevated levels of beta‐defensin‐2 and beta‐defensin‐3, correlating with increased tumor cell apoptosis and suppressed tumor progression [43].

### 5.2 Innate Immune Activation

BCG therapy also activates the innate immune system to combat tumors. BCG activates Toll‐like receptors (TLR2/4/9) on bladder epithelial cells and macrophages, inducing cytokine and chemokine release to attract immune cells to the tumor microenvironment [44]. Natural killer (NK) cells are markedly activated during BCG treatment. BCG increases NKG2D ligand expression on tumor cells, boosting NK cell cytotoxicity. In murine bladder cancer models, BCG significantly increases NK cell numbers and activity, leading to effective tumor cell elimination and metastasis suppression [45]. Neutrophils are also recruited to tumor sites during BCG therapy. These cells generate nitric oxide (NO) and reactive oxygen species (ROS), which directly kill tumor cells. Furthermore, ROS and NO induce apoptosis and inhibit angiogenesis, further restricting tumor growth [46].

### 5.3 Adaptive Immune Response

BCG activates adaptive immunity by promoting dendritic cell‐mediated antigen presentation, which stimulates CD4^+^ Th1 cells to secrete interferon‐gamma (IFN‐γ). This cytokine enhances immune cell activity and promotes tumor cell apoptosis [45]. CD8^+^ T cells are also activated during BCG therapy, enabling recognition and targeted destruction of tumor‐specific antigens like MAGE‐A3. Clinical studies report increased CD8^+^ T cell activity in BCG‐treated patients, correlating with tumor regression and reduced recurrence [47]. BCG also induces immunological memory, providing long‐term protection against tumor relapse. Long‐term follow‐up studies show durable remission in BCG‐treated patients, underscoring its capacity to establish persistent immune memory [47].

### 5.4 Tumor Microenvironment Remodeling

BCG reshapes the tumor microenvironment by altering bladder microbiota composition, enhancing immune surveillance. For example, BCG enriches beneficial bacterial populations that produce antimicrobial compounds and modulate immune responses [46]. Macrophages polarized to the M1 phenotype (pro‐inflammatory) during BCG therapy secrete cytotoxic mediators and pro‐inflammatory cytokines, directly killing tumor cells. Murine models confirm that BCG increases M1 macrophage activity, significantly suppressing tumor growth [48]. BCG also downregulates the antiapoptotic protein Mcl‐1 in tumor cells, sensitizing them to immune‐mediated destruction [48].

### 5.5 Cytokine Network Synergy

BCG triggers a cytokine storm characterized by the robust production of pro‐inflammatory cytokines like TNF‐α, IL‐12, IL‐2, and IFN‐γ. These cytokines form autocrine/paracrine signaling networks that sustain immune activation [49].

IL‐12 and IL‐2 drive T cell proliferation and activation, while IFN‐γ enhances macrophage and NK cell cytotoxicity [45]. TNF‐α induces tumor cell apoptosis and inhibits angiogenesis. Clinical studies confirm elevated levels of these cytokines in BCG‐treated patients, correlating with tumor suppression and improved outcomes [50]. This comprehensive immunomodulatory framework underpins BCG's efficacy as a first‐line immunotherapy for NMIBC.

**FIGURE 1:**
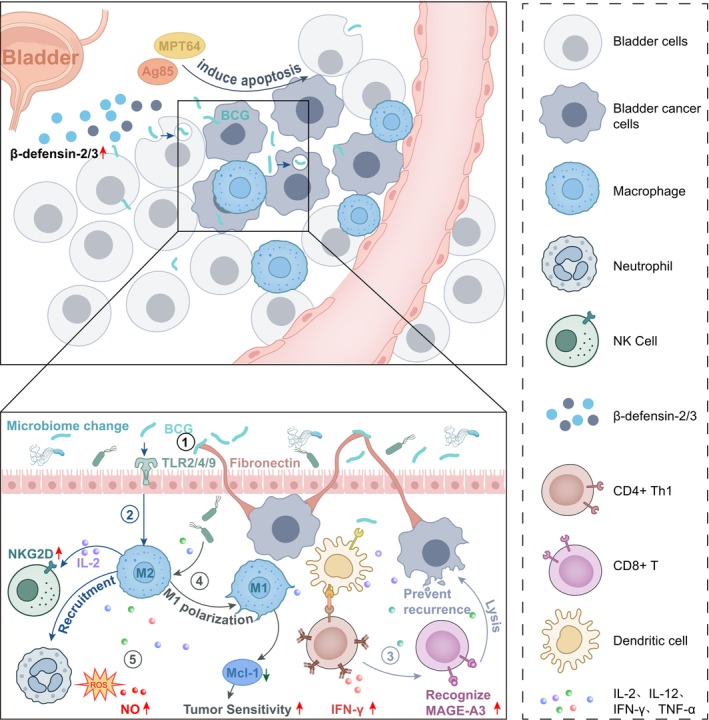
BCG Mechanism in Bladder Cancer Treatment. The mechanism of BCG in the treatment of bladder cancer involves several key aspects: ① The BCG treatment mechanism for bladder cancer encompasses several critical components. BCG demonstrates a direct toxic impact on bladder cancer cells; ② it shows that BCG triggers innate immune responses; ③ it highlights the role of BCG in stimulating adaptive immune responses; ④ it clarifies the mechanisms by which BCG influences the microbiome to stimulate innate and adaptive immunity. ⑤ In the bladder microenvironment, BCG induces the production of pro‐inflammatory cytokines like TNF‐α, IL‐2, and IFN‐γ, which enhance tumor sensitivity and lower the risk of bladder cancer recurrence. The cellular and molecular elements are created with BioRender.com.

BCG's anticancer effects are varied, including direct cytotoxicity, immune activation, and reprogramming of the tumor microenvironment. These processes collectively establish a robust immunological attack against bladder cancer. Future research should aim to clarify the spatiotemporal interactions among immune subsets to enhance BCG efficacy and inform the development of rational combination therapies (Figure 1).

## 6 Shortage of BCG and Its Influence in Clinical

A 2019 study in Urology noted a continual increase among patients receiving BCG treatment, particularly those with high‐grade T1 tumors and CIS, despite its long‐standing availability [51]. Production has lagged behind demand due to factors like plant shutdowns from potential batch contamination, leading to a BCG supply shortage [52]. Physicians treating NMIBC patients have faced significant challenges over the past 6 years due to global BCG shortages [53]. Despite the challenges in producing BCG, it remains irreplaceable, necessitating its continued production by all available means. The BCG shortage has prompted a reevaluation of existing treatment guidelines for its rationed administration. Potential schedule adjustments include shortening maintenance treatment duration, lowering the BCG dose in intravesical instillations, or utilizing alternative BCG substrains. These strategies are deemed valuable during BCG shortages. Furthermore, the limited availability of BCG has underscored the necessity of identifying potential therapeutic alternatives for affected patients [54]. While few alternatives have been explicitly developed as full replacements for BCG intravesical instillations, numerous strategies are under investigation to either improve its effectiveness or combine it with other chemotherapeutic/immunotherapeutic agents for enhanced outcomes [15]. These novel approaches are primarily being tested in BCG‐unresponsive patients, as detailed in the following section.

Key recommendations to mitigate BCG shortages are outlined below [55]. For patients with intermediate‐risk NMIBC, prioritize intravesical chemotherapy (e.g., mitomycin, epirubicin, or gemcitabine) over BCG as first‐line therapy. Administer induction weekly for 6 to 8 weeks, followed by monthly maintenance for 1 year. For second‐line treatment, a one‐third BCG dose may substitute the full dose. Grouping three patients for same‐day treatment reduces BCG waste, and maintenance BCG can be omitted.

For high‐risk NMIBC, Low‐tier high‐risk tumors (TaHG): Shorten maintenance BCG to 1 year, using reduced doses for both induction and maintenance [56]. Alternatives include mitomycin C (up to 1 year) or electromotive mitomycin (EMDA‐MMC) [54]. Gemcitabine, epirubicin, or sequential gemcitabine/docetaxel are additional options [57]. Cystectomy is generally recommended as a standard treatment option for patients with high‐risk T1 high‐grade (HG) tumors and CIS [55].

## 7 Options for BCG‐Unresponsive Patients

### 7.1 Definition of BCG‐Unresponsive

BCG therapy demonstrates limited efficacy in 30%–50% of patients with high‐risk NMIBC, with high‐grade recurrence representing a significant clinical challenge [58]. The interpretation of optimal management strategies is complicated by historical inconsistencies in defining BCG failure. Technically, BCG failure encompasses any high‐grade recurrence occurring during or following BCG therapy, but stratification based on recurrence timing and prior treatment adequacy is critical for clinical decision‐making [59].

According to the EAU Guidelines and FDA criteria, BCG‐unresponsive disease is strictly defined in patients who have received adequate BCG therapy (completion of ≥ 5/6 induction doses plus ≥ 2/3 maintenance doses or a second induction course) and exhibit one of the following: persistent T1 HG tumor at 3 months; recurrent Ta HG tumor within 6 months of treatment completion; persistent/recurrent CIS within 12 months; or HG tumor development during maintenance therapy. In contrast, BCG‐relapsing disease refers to high‐grade recurrences that do not meet these criteria (e.g., Ta/T1 HG recurrence > 6 months or CIS > 12 months after adequate BCG) [11, 12].

This stratification has profound clinical implications. BCG‐relapsing patients may retain sensitivity to salvage intravesical therapies, including repeat BCG instillation, and generally exhibit more favorable oncological outcomes [60]. Conversely, BCG‐unresponsive NMIBC patients derive minimal benefit from additional BCG immunotherapy and face heightened progression risk. For this subgroup, radical cystectomy remains the standard of care, though bladder‐preserving alternatives (e.g., device‐assisted intravesical chemotherapy, immune checkpoint inhibitors) are emerging as options for patients unfit for surgery, preferably within clinical trial settings [61]. Clear delineation between these entities is essential for therapeutic optimization and protocol standardization in clinical research, ensuring appropriate patient stratification for novel interventions.

### 7.2 Recommended Treatments for BCG‐Unresponsive Patients

For these patients, the typical treatment is radical cystectomy; however, due to the risk of perioperative complications and morbidity, many opt to delay this procedure until all other options are exhausted, although it might progress to muscle‐invasive or metastatic disease [62]. Regrettably, no standard salvage therapies exist for BCG‐unresponsive disease at present. Certain intravesical chemotherapy (mitomycin c, gemcitabine, epirubicin, valrubicin, and docetaxel) and checkpoint inhibitor (nivolumab and recently approved pembrolizumab) can be considered as acceptable alternative therapies [63, 64]. Research indicates that chemotherapy can prevent recurrence, but it does not halt progression. Salvage intravesical chemotherapy in BCG‐unresponsive patients is suboptimal, with about 70% to 80% experiencing recurrence within 2 years of treatment initiation [65]. It is crucial to recognize that all approved salvage intravesical therapies for BCG‐unresponsive cases are oncologically inferior to cystectomy [66].

New bladder‐preserving therapies are in significant demand because salvage intravesical chemotherapy is not very effective for BCG failure. Patients ineligible or unwilling to undergo cystectomy may participate in trials of new intravesical therapies for BCG‐unresponsive disease, aiming to lower recurrence rates and delay or prevent progression to muscle‐invasive disease [67].

## 8 Respresentative Clinical Trials for BCG‐Unresponsive Patients

The landscape of treatment for BCG‐unresponsive NMIBC is rapidly evolving, with promising results from clinical trials across multiple therapeutic modalities. Targeted therapies, including immune checkpoint inhibitors and FGFR inhibitors, have demonstrated durable responses and favorable safety profiles, establishing new standards of care for select patient populations. Device‐assisted approaches, such as hyperthermic intravesical chemotherapy and sustained drug‐release systems, have improved local drug delivery and efficacy, while gene therapies and oncolytic viruses have shown potential in activating antitumor immunity and inducing tumor‐specific cell death (Table 2).

**TABLE 2:** Key clinical trials of BCG‐unresponsive patients.

Therapy type	Agent/Intervention	Target/Mechanism	Stage/Approval status	Key outcomes
Immune checkpoint inhibitors	Pembrolizumab	PD‐1	FDA‐approved (2020)	CRR: 41% (CIS ± papillary tumors); Median duration of response: 16.2 months
	Anktiva (ALT‐803) + BCG	IL‐15 receptor	FDA‐approved (2024)	CRR: 71% (CIS); Median duration of response: 26.6 months; 24‐month PFS: 96%
Targeted therapy	Erdafitinib	FGFR1‐4	Phase II	CRR: 100% (first evaluation), 75% (second evaluation); Median response duration: 3.0 months
	Vicinium (Oportuzumab monatox)	EpCAM	Phase III	CRR (CIS): 40% at 3 months; 52% of responders maintained disease‐free status at 12 months
Gene therapy	Nadofaragene firadenovec	IFN‐α2b gene delivery	FDA‐approved (2022)	CRR: 51% at 3 months; Median duration of response: 9.7 months; 46% of responders maintained response for ≥ 12 months
	CG0070	GM‐CSF‐expressing oncolytic virus	Phase III	CRR: 75% at any time; 46% maintained CR at 12 months; Durable responses up to 21 months
Device‐assisted chemotherapy	CHT‐MMC	Hyperthermia + Mitomycin C	Phase III	24‐month recurrence‐free survival: 57.4% (BCG‐unresponsive); Progression‐free survival: 90.1%
	TAR‐200	Sustained gemcitabine release	FDA priority review (2025)	CRR: 82.4%; 52.9% of responders remained cancer‐free for ≥ 1 year
Photodynamic therapy	TLD1433‐PDT	Ru (II) polypyridyl complex	Phase 1b	CRR: 67% (2/3 patients at therapeutic dose); Durable response at 18 months
Gene therapy (Emerging)	BC‐819 (DTA‐H19)	H19 promoter‐driven toxin	Phase II (CODE trial)	Proof‐of‐concept for tumor‐specific apoptosis; CRR at 12 weeks not disclosed
ADC (Emerging)	Disitamab vedotin + Tislelizumab	HER2	Phase II (TRUCE‐04, ongoing)	Results pending; Enrollment: 176 patients

Abbreviations: ADC, antibody‐drug conjugate; CIS, carcinoma in situ; CRR, complete response rate; NMIBC, nonmuscle invasive bladder cancer; PFS, progression‐free survival.

### 8.1 Targeted Therapy

#### 8.1.1 Inhibition of the PD‐1/PD‐L1 Pathway

Immune checkpoint inhibitors targeting PD‐1/PD‐L1 have revolutionized metastatic bladder cancer treatment by blocking inhibitory signals that suppress T‐cell activity, enabling immune‐mediated tumor destruction [68, 69, 70]. Research shows BCG regulates PD‐L1 expression via the MAPK pathway, and combining BCG with anti‐PD‐L1 therapies enhances CD8+ T‐cell activity, particularly in BCG‐unresponsive patients [71, 72, 73, 74].

In 2020, the FDA approved pembrolizumab for high‐risk, BCG‐unresponsive NMIBC based on the KEYNOTE‐057 trial (NCT02625961) [75]. Data from cohort A (patients with CIS with or without papillary tumors) of this trial showed that pembrolizumab monotherapy achieved a 3‐month complete response rate of 41%, with the complete response rate reaching 57% in patients with high PD‐L1 expression, and the median duration of response was 16.2 months. In terms of safety, 15% of patients experienced grade 3 treatment‐related adverse events, the most common of which included colitis and diarrhea. Compared with the 23% response rate of historical control with gemcitabine monotherapy, pembrolizumab showed superior clinical efficacy and has become the new standard treatment option for this patient population [76].

Ongoing trials like KEYNOTE‐676 (NCT03711032, Phase III) explore pembrolizumab‐BCG combinations for recurrent or high‐risk NMIBC [77, 78, 79]. Other agents, including atezolizumab [80, 81] (26% response in PD‐L1‐high metastatic cases), nivolumab [82, 83, 84], and the investigational sasanlimab [85], are being tested in advanced trials (Phases Ib–III) as monotherapies or combined with BCG, with completion dates extending to 2027. These studies aim to optimize efficacy and safety, emphasizing PD‐L1 as a predictive biomarker and underscoring combination immunotherapy as the future paradigm for bladder cancer treatment.

#### 8.1.2 IDO Inhibitors

Indoleamine 2,3‐dioxygenase (IDO) [86] is a key immunosuppressive enzyme in cancer, suppressing CD8^+^ T cells and natural killer (NK) cells while promoting regulatory T cells (Tregs) and myeloid‐derived suppressor cells (MDSCs). It operates via tryptophan depletion, inhibiting mTORC1 and protein kinase C theta, thereby dampening T‐cell activity. IDO1, its isoform, is overexpressed in bladder cancer, highlighting its potential as a therapeutic target. Indoximod, epacadostat, and navoximod were first to be evaluated as IDO inhibitors in clinical trials. Some of the tested compounds are used alone or in combination with immunotherapy (CTLA4, PD‐1 blockade), chemotherapy, COX‐2 inhibitors (celecoxib), and membrane‐associated PGE2 synthase inhibitors (MF63) [87].

BMS‐986205 (linrodostat), an IDO1 inhibitor, was evaluated in combination with nivolumab for BCG‐unresponsive NMIBC in the CheckMate 9UT trial (NCT03519256), which was terminated due to insufficient accrual [15]. Additionally, BMS‐986205 is being investigated in the Phase III ENERGIZE trial (NCT03661320) in combination with nivolumab and neoadjuvant chemotherapy for MIBC [88].

#### 8.1.3 Fusion‐Proteins

ALT‐801 is a bifunctional fusion protein combining IL‐2 with a T‐cell receptor (TCR) domain targeting the p53 antigen peptide (aa264‐272) on cancer cells. It activates lymphocytes similarly to IL‐2 but enhances antitumor activity by promoting NK cell infiltration into tumors and directing immune cells to tumors via peptide/HLA complexes [89]. The Phase Ib/II study (NCT01625260) evaluated ALT‐801 combined with gemcitabine in 12 patients with BCG‐refractory non‐pathological complete response muscle invasive bladder cancer ineligible for radical cystectomy. This multicenter, nonrandomized trial was terminated prematurely but demonstrated promising results: in the 0.06 mg/kg cohort, 50% (3/6) of evaluable patients achieved biopsy‐proven complete responses, with 2 responses lasting ≥ 18 months. Safety assessment revealed hepatotoxicity as the primary dose‐limiting toxicity (Grade ≥ 3 in 1/6 patients), along with manageable adverse events including fatigue, chills, pruritus, rash, anorexia, and edema, with no hematologic toxicities. Immunological monitoring showed transient IFN‐γ and IL‐6 induction postdosing without significant TNF‐α or IL‐10 response. While the study met its primary safety endpoints over 12 weeks, secondary efficacy measures (duration of response, progression‐free survival, event‐free survival, and overall survival up to 3 years) suggested durable clinical activity, supporting ALT‐801's potential in this challenging patient population [90].

ALT‐803 is an IL‐15 superagonist complex composed of the IL‐15 N72D mutant and the dimer IL‐15 receptor α Su/IgG1 Fc fusion protein. It enhances the tumor killing activity of NK and CD8^+^ T cells and has a longer half‐life than recombinant IL‐15. Clinically, in the Phase 2/3 trial (QUILT‐3.032, NCT03022825) of intravesical ALT‐803 combined with BCG for BCG‐unresponsive NMIBC, the recommended dose of ALT‐803 and BCG was 400 μg/instillation and 50 mg/instillation, respectively. Approximately 71% of patients with CIS achieved a complete response at any time, with a median duration of 26.6 months. About 96% of patients with CIS had a 24‐month survival rate without progression to MIBC, and 91% avoided cystectomy within 24 months. For patients with papillary disease, the 12‐month and 24‐month disease‐free survival rates were 57% and 48%, respectively, and 95% avoided cystectomy. The overall 24‐month survival rate of 160 patients was 99%. In clinical trials, the most common adverse events were grades 1 and 2, including dysuria (22%), hematuria (18%), pollakiuria (19%), urinary urgency (12%), and fatigue (16%), with no serious treatment‐related toxicities, grade 4 or 5 adverse events, or immunogen‐related side effects [91, 92].

Anktiva (nogapendekin alfa inbakicept‐pmln), a first‐in‐class IL‐15 superagonist fusion protein, was FDA‐approved in April 2024 for intravesical use with BCG in adults with BCG‐unresponsive NMIBC (CIS ± papillary tumors). The complex comprises IL‐15N72D (high‐affinity IL‐15 mutant) and dimeric IL‐15Rα sushi domain/IgG1 Fc fusion protein, selectively activating CD8^+^ T, NK, and memory T cells via IL‐15Rα/CD122/CD132 without stimulating Treg expansion. In the phase 2/3 QUILT‐3.032 trial, 71% of evaluable BCG‐unresponsive NMIBC/CIS patients achieved complete response (CR) with median CR duration of 26.6 months; 24‐month progression‐free and overall survival rates were 84.7% and 94.3%, respectively, with only 7% of CR patients requiring cystectomy. The combination demonstrated acceptable safety, predominantly grade 1/2 adverse events (dysuria, haematuria, urinary frequency). As a novel antibody‐cytokine fusion, Anktiva synergizes with BCG to enhance antitumor immunity, providing a bladder‐preserving option for BCG‐unresponsive NMIBC and reshaping urothelial carcinoma treatment [93, 94].

#### 8.1.4 Targeted Toxin Drugs

Vicinium (oportuzumab monatox; VB4–845) is a recombinant protein. It fuses a single‐chain variable fragment of a humanized epithelial cell adhesion molecule (EpCAM) antibody with Pseudomonas exotoxin A (PE) [95]. The tumor‐targeting specificity of Vicinium is due to the high‐level expression of the EpCAM surface marker on the plasma membrane of urothelial carcinoma cells [96, 97, 98]. The anti‐EpCAM part of Vicinium binds to EpCAM, which initiates receptor‐mediated endocytosis and internalization of the Pseudomonas exotoxin. Once inside the cell, PE is cleaved into fragments with molecular weights of 37,000 and 27,000. The larger fragment enters the Golgi apparatus and interacts with elongation factor 2 (EF). This interaction, along with glycosylation, leads to the inhibition of protein synthesis and eventually induces apoptosis.

Vicinium's efficacy is restricted to EpCAM‐expressing tumors, and its clinical trials consistently require confirmed EpCAM expression as an enrollment criterion. A phase III single‐arm trial (NCT02449239) in BCG‐unresponsive NMIBC demonstrated a 3‐month recurrence‐free survival rate of 71% in patients with papillary tumors, which declined to 50% at 12 months. For patients with CIS, the 3‐month complete response rate was 40%, and 52% of responders maintained disease‐free status at 12 months. Overall, the median time to cystectomy was significantly prolonged in 3‐month responders (34.0 months) compared to nonresponders (20.7 months), with treatment‐related adverse events predominantly grade 1–2 (52%) [99, 100].

Disitamab vedotin (RC48) is a HER2‐targeted antibody‐drug conjugate consisting of a HER2‐specific antibody, monomethyl auristatin E (MMAE), and a cleavable valine‐citrulline linker, which can selectively bind to HER2‐expressing tumor cells, internalize, and release MMAE intracellularly to exert cytotoxic effects including bystander killing of adjacent heterogeneous tumor cells. The ongoing phase II TRUCE‐04 trial (NCT05495724) is investigating disitamab vedotin in combination with tislelizumab for HER2‐positive high‐risk NMIBC in a nonrandomized parallel study design with planned enrollment of 176 patients. This chemo‐free regimen aims to provide an alternative treatment option, particularly for patients who are not candidates for complete resection, building on prior data showing promising response rates and tolerable toxicity profiles of RC48‐based therapies, though full clinical outcomes are still pending [101].

#### 8.1.5 TLR Agonist

Toll‐like receptor (TLR) is a pattern‐recognition receptor that plays a key role in the innate immune response. It mainly functions by recognizing diverse pathogen‐associated molecular patterns [102]. When TLR on tumor cells is activated, it sets off the NF–κB pathway. This activation results in the production of cytokines and chemokines, which in turn have an impact on tumor growth, immune tolerance, and angiogenesis.

Conversely, on dendritic cells, TLR is predominantly present in the forms of TLR3 and TLR4. These TLRs influence the activation, maturation, and migration of dendritic cells. Mature dendritic cells can initiate antitumor immune responses through Th1‐induced cytotoxicity [103]. NMIBC shows a moderate level of expression of TLR2, 3, 4, 5, 7, and 9. The application of TLR agonists targeting these TLRs generates a moderate antitumor response. Clinically, it is more advisable to use TLR agonists in combination with BCG rather than as a single‐agent therapy [104]. TARA‐002 is an investigational inactivated *Streptococcus pyogenes* ‐treated cellular therapy that targets TLR4. The ADVANCED‐2 study (NCT05951179) is an ongoing open‐label Phase II trial evaluating intravesical TARA‐002 (40 KE), a lyophilized *Streptococcus pyogenes* preparation, in adults ≥ 18 with high‐grade NMIBC (CIS ± Ta/T1). It includes two cohorts: Cohort A (BCG‐naïve or BCG‐exposed ≥ 24 months from CIS diagnosis) and Cohort B (BCG‐unresponsive within 12 months of adequate therapy), with a target enrollment of 127 subjects. Treatment consists of induction (6 weekly doses), reinduction (for persistent disease at 3 months), maintenance (3 weekly doses at 3, 6, 9, 12, 15, 18 months), and 60‐month follow‐up. As of October 18, 2024, 23 subjects were enrolled (median age 74; 95.7% White non‐Hispanic; 78.3% male). Baseline ECOG scores were 0 (78.3%) or 1 (21.7%). Disease characteristics: CIS only (56.5%), CIS + Ta (26.1%), CIS + T1 (17.4%). Among 20 evaluable subjects, the high‐grade complete response rate at any time was 60% (12/20). All 23 received ≥ 1 TARA‐002 dose; 15 (65.2%) had TEAEs, 5 with drug‐related TEAEs (all Grade 1). No drug‐related SAEs, TEAEs leading to withdrawal, or deaths occurred. Common AEs included bladder spasm, burning sensation, and UTI. Preliminary data show TARA‐002 is well‐tolerated with encouraging antitumor activity in BCG‐naïve, exposed, and unresponsive CIS ± Ta/T1 NMIBC subjects [105, 106].

#### 8.1.6 MetAP2 Inhibitors

Methionine aminopeptidase 2 (MetAP2) is a metalloproteinase composed of 478 amino acids. Its function involves removing the N‐terminal methionine from newly synthesized proteins, which is of great significance in tumorigenesis and angiogenesis [107]. Consequently, MetAP2 inhibitors have attracted extensive attention, and research on them is advancing.

APL‐1202 is a reversible oral MetAP2 inhibitor with antiangiogenic and antitumor activities. Its Phase II ANTICIPATE trial (NCT04813107) evaluated neoadjuvant APL‐1202 plus tislelizumab versus tislelizumab alone in cisplatin‐ineligible/refractory muscle‐invasive bladder cancer patients. Interim analysis of 32 evaluable patients showed the pathological complete response (pCR) rate was 39% (7/18) in the combination group versus 21% (3/14) in the single‐agent group. The < pT2N0 rate was 44% (8/18) versus 21% (3/14). TEAEs occurred in 94.4% (17/18) of combination group patients and 78.6% (11/14) of single‐agent group patients. Common grade ≥ 3 TEAEs included anemia (22.2%) and lymphocyte count decrease (16.7%) in the combination group, and intestinal obstruction (21.4%) in the single‐agent group. Drug discontinuation due to AEs occurred in 16.7% (3/18) and 14.3% (2/14) of patients, respectively, with no treatment‐related deaths [108]. Currently, a Phase III randomized controlled trial (NCT04736394) is recruiting 800 BCG‐naïve patients with intermediate‐risk NMIBC to evaluate the safety and efficacy of oral APL‐1202 versus intravesical Epirubicin Hydrochloride. The study, which started on September 29, 2021, and is expected to complete by December 31, 2025, primarily measures event‐free survival (EFS) up to 48 months through independent pathological review. Secondary endpoints include overall survival, recurrence‐free rates (at 12, 18, and 24 months), progression‐free rates, clinical benefit rates (defined as pathological improvement without high‐risk recurrence), and quality of life assessments using EORTC‐QLQ‐C30 and NMIBC24 questionnaires. The study also tracks cystectomy‐free survival as an additional outcome measure. This multicenter trial will provide comprehensive data on both treatment efficacy and patient‐reported outcomes for these two therapeutic approaches in intermediate‐risk NMIBC [109].

#### 8.1.7 FGFR‐Targeted Therapies

Fibroblast growth factor receptors (FGFRs) belong to a family of receptor tyrosine kinases. Comprising four isoforms—FGFR1, FGFR2, FGFR3, and FGFR4—they are situated on the cell membrane. These receptors play a vital role in various cellular processes, including cell proliferation, differentiation, migration, angiogenesis, and the maintenance of metabolic homeostasis [110]. In NMIBC, FGFR3 mutations are common. Variants such as S248C often arise from APOBEC‐mediated mechanisms. Roughly 65% of NMIBC patients have FGFR3 mutations [111].

Erdafitinib (Balversa) is an oral pan‐FGFR inhibitor targeting FGFR1–4. FDA‐approved for locally advanced/metastatic urothelial carcinoma with susceptible FGFR2/3 alterations (mutations or fusions) after platinum‐based chemotherapy [112]. In the THOR‐2 (NCT04172675) study, 10 BCG‐unresponsive high‐risk NMIBC patients with FGFR alterations received erdafitinib: CR rates 100% (first evaluation) and 75% (second evaluation), median response duration 3.0 months. Common TEAEs: dry mouth, hyperphosphatemia, dysgeusia, diarrhea. One patient had Grade 2 retinal detachment (discontinued treatment), 1 had Grade 1 subretinal fluid; both resolved. Grade ≥ 3 treatment‐related TEAEs in 3 patients; 1 had serious treatment‐related TEAEs, 1 discontinued due to treatment‐related TEAE; no treatment‐related deaths. Additionally, erdafitinib significantly prolonged recurrence‐free survival versus intravesical chemotherapy in BCG‐recurrent, FGFR‐altered high‐risk NMIBC patients ineligible/refusing cystectomy (median not reached vs. 11.6 months; HR = 0.28, 95% CI 0.1–0.6, *p* = 0.0008) [113].

Rogaratinib is a pan‐FGFR inhibitor targeting FGFR1‐4 [111]. A phase II study (NCT04040725) was designed for BCG‐refractory high‐risk NMIBC with FGFR1/2 overexpression, but it was ultimately withdrawn and failed to complete enrollment. In other phase II trials for urothelial carcinoma, rogaratinib showed an objective response rate of 21%, with a median progression‐free survival of 2.7 months and overall survival of 8.3 months (similar to chemotherapy) [114].

### 8.2 Device‐Assisted Chemotherapy

#### 8.2.1 Chemotherapy Enhancement Technology

Device‐assisted chemotherapy aims to improve drug penetration through the urothelium. It achieves this by leveraging heat (chemohyperthermia, CHT) or electrical current (electromotive drug administration, EMDA) [115].

Elevated temperatures can improve blood perfusion and increase cell permeability [116].

In a large multicenter prospective study of 835 patients with NMIBC treated with CHT using the Combat bladder recirculation system and Mitomycin C (MMC), the 24‐month recurrence‐free survival (RFS) was 70.0% overall, with 75.0% for BCG‐naïve patients and 57.4% for BCG‐unresponsive patients (64.5% in those with papillary‐only disease vs. 43.6% in those with CIS ± papillary disease). The 24‐month progression‐free survival (PFS) was 90.8% overall, with 91.3% for BCG‐naïve and 90.1% for BCG‐unresponsive cohorts. Treatment was well‐tolerated, with 68.1% of patients experiencing no adverse events, 25.6% reporting minor (Grade 1–2) events, and only 2.0% experiencing severe (Grade 3) adverse events, while only 5% of patients required treatment discontinuation due to adverse effects [117]. In a study of 59 patients with BCG‐unresponsive or intolerant high‐grade T1 nonmuscle‐invasive bladder cancer treated with intravesical CHT‐MMC (42°C ± 2°C bladder wall hyperthermia plus 20 mg/50 mL mitomycin C instilled twice at 30‐min intervals), recurrence‐free survival rates were 58.7% at 24 months and 48% at 44 months, with progression‐free survival rates of 72.6% and 66.2%, respectively, over the same periods. Subgroup analysis showed significantly higher recurrence‐free survival in patients with single tumors (81.8%) versus 2–5 tumors (48.2%) or multiple tumors (> 5, 11%) at 44 months, while those receiving 12 treatment sessions (induction plus maintenance) demonstrated superior recurrence‐free (64.9% vs. 33.2%) and progression‐free (80.5% vs. 55.3%) survival compared to 6 sessions. Treatment was well‐tolerated, with increased dysuria and spasms reported but no discontinuations required, and 5‐year overall survival reached 88.8% [118].

EMDA is thought to boost drug delivery across biological membranes. This results in a higher accumulation of the drug in bladder tissue. EMDA increases MMC penetration via electrical current, achieving 30‐fold higher urothelial concentrations than passive diffusion. In a phase III RCT with 86‐month median follow‐up, neoadjuvant EMDA‐MMC before TURBT resulted in a 62% recurrence‐free rate, significantly higher than TURBT alone (36%) or passive MMC (41%). Median time to recurrence was 52 months with EMDA‐MMC versus 12 months (TURBT alone) and 16 months (passive MMC). For BCG‐unresponsive high‐risk NMIBC, the 3‐year progression‐free rate was 61.5%, with 61.5% of patients avoiding radical cystectomy. Common adverse events: irritative voiding (42%), bladder spasms (33%), pain (30%); 2% discontinued due to catheter intolerance. Mild skin erythema at electrode sites is specific to EMDA [119].

The Synergo system (radiofrequency‐induced thermochemotherapy, RITE) uses a catheter with an integrated radiofrequency antenna to maintain bladder wall temperatures at 42°C ± 2°C while circulating MMC via a cooled circuit [120]. In the RITE‐USA clinical trial (NCT03335059) for BCG‐unresponsive nonmuscle invasive bladder cancer patients, the complete response rate (CRR) was evaluated as the primary outcome, requiring absence of CIS, high‐grade papillary tumors, T1 tumors, extravesical UC tumors, and disease progression at 3 months posttreatment. Secondary outcomes measured disease‐free duration in complete responders, with follow‐up continuing for up to 33 months. However, the study was terminated early after enrolling only five patients, limiting the ability to draw definitive conclusions about efficacy and safety [121].

External Beam Radiation Therapy (EBRT) is a device‐assisted method that enhances the efficacy of chemotherapy or immunotherapy through radiation equipment. In the multiarm, multicenter, phase 1 ADAPT‐BLADDER study (NCT03317158), EBRT has been combined with immune checkpoint inhibitors such as durvalumab to improve the control of BCG‐unresponsive NMIBC. A clinical trial evaluated durvalumab (an anti‐PD‐L1 antibody) in combination with EBRT for patients with BCG‐unresponsive NMIBC. In this multicenter phase 1 trial, patients in the D + EBRT cohort (*n* = 12) received 1120 mg of durvalumab intravenously every 3 weeks for eight cycles, with concurrent EBRT (6 Gy × 3 in cycle 1 only). The recommended phase 2 dose (RP2D) for the D + EBRT regimen was determined to be full‐dose durvalumab and 6 Gy fractions × 3. Regarding safety, 42% of patients in the D + EBRT cohort experienced grade 3–4 adverse events, including pneumonitis, maculopapular rash, elevated ALT, elevated AST, and elevated lipase, with one patient experiencing a grade 3 dose‐limiting toxicity event of autoimmune hepatitis. Efficacy outcomes showed that the 3‐month complete response (CR) rate was 50%, the 6‐month CR rate was 33%, and the 12‐month CR rate was 33% in the D + EBRT cohort [122].

The COMBAT system delivers hyperthermic intravesical chemotherapy (HIVEC) with MMC at 41°C–43°C, involving an induction course of 6 weekly sessions followed by a maintenance course of 6 monthly sessions. In a retrospective single‐center study of 51 patients with high‐risk NMIBC, including 55% BCG‐unresponsive cases, the 1‐year and 2‐year recurrence‐free survival (RFS) were 67% and 40% respectively, with a 2‐year progression‐free survival (PFS) of 98% and a bladder preservation rate of approximately 77%. Notably, no statistically significant differences in RFS were observed between BCG‐unresponsive and BCG‐naive patients, or between those with and without carcinoma in situ (CIS). HIVEC was well‐tolerated, with no grade 3 or 4 adverse events reported; only 2 out of 51 patients discontinued therapy due to adverse events (refractory bladder spasms and MMC‐induced pneumonitis), and the most common side effects included spasms (15.6%), rash (7.8%), and hematuria (3.9%) [123].

#### 8.2.2 Photodynamic Therapy

Photodynamic therapy serves as an effective option for bladder cancer patients who are either unable or reluctant to undergo surgery. It can reduce recurrence rates and relieve symptoms, thus enhancing the quality of life for those with advanced‐stage bladder cancer [124]. This therapy utilizes light energy to eradicate malignant epithelial cells in the urinary tract. When the photosensitizer (PS) absorbs light energy, it shifts from its ground state to an excited state. This enables the transfer of energy to surrounding oxygen molecules, generating cytotoxic reactive oxygen species that target tumor cells. The PS mainly reaches tumor cells through diffusion in the bladder lumen. By injecting or orally administering the PS to the patient, it can be selectively taken up by tumor cells and accumulate in them after a specific period. Current research focuses on systems where a photosensitizing agent is administered intravesically, followed by using a urinary catheter to deliver light from an external source.

TLD1433, a novel Ru (II) polypyridyl complex photosensitizer, shows 200‐fold greater selectivity for bladder cancer cells compared to healthy bladder tissue. This characteristic has sparked significant interest in photodynamic therapy (PDT) [125]. In March 2017, a Phase 1b study was launched to evaluate TLD‐1433‐mediated PDT in BCG‐unresponsive patients with NMIBC. Six BCG‐unresponsive patients were enrolled in an open‐label, single‐arm, dose‐escalating study of PDT. TLD‐1433 was instilled intravesically for 60 min preoperatively. PDT was performed under general anesthesia using intravesically delivered irradiation of the bladder wall with green light (520 nm) to a dose of 90 J/cm^2^. The study concluded that PDT with TLD‐1433 is safe for treating BCG‐unresponsive nonmuscle‐invasive bladder cancer. All adverse events were of grade ≤ 2, with the most common being lower urinary tract symptoms that improved significantly within the first 90 days and mostly resolved within 180 days. No serious adverse events or photosensitivity reactions occurred. TLD‐1433 was cleared in urine within 24 h and plasma within 72 h. Of three patients treated at the therapeutic dose (0.70 mg/cm^2^ bladder surface area), two achieved a complete response at 180 days, which was durable at 18 months. A larger Phase 2 trial is planned to further assess TLD‐1433 [126].

#### 8.2.3 Drug‐Releasing Implants

TAR‐200 is an innovative intravesical drug‐delivery system engineered to enable sustained local release of gemcitabine directly within the bladder, addressing the limitations of conventional instillation therapies such as low bladder permeability and drug washout due to frequent urination [127]. It has been accepted for priority review by the US FDA to treat high‐risk NMIBC, specifically for patients with BCG‐unresponsive high‐risk NMIBC with carcinoma in situ, with or without papillary tumors [128]. The new drug application was supported by results from the phase 2b SunRISe‐1 trial (NCT04640623), in which TAR‐200 monotherapy was associated with a complete response (CR) rate of 82.4%, with 52.9% of patients remaining cancer‐free for at least 1 year after achieving a CR. The system is placed into the bladder by a healthcare professional using a copackaged urinary placement catheter in an outpatient setting in under 5 min. TAR‐200 exhibits a favorable safety profile, with most treatment‐emergent adverse events being mild to moderate in severity, such as pollakiuria and dysuria [129]. In the ongoing phase III clinical trial (NCT06211764), TAR‐200 is being evaluated in patients with recurrent high‐risk NMIBC who have previously received BCG therapy and are either ineligible for or unwilling to undergo radical cystectomy [130]. This trial compares TAR‐200, administered as an induction phase every 3 weeks followed by a maintenance phase every 12 weeks, with investigator‐chosen intravesical chemotherapy (either mitomycin C or gemcitabine). Additionally, TAR‐200 exhibits a favorable safety profile, with most treatment‐emergent adverse events being mild to moderate in severity, such as pollakiuria and dysuria. These preliminary results suggest that TAR‐200 holds potential for achieving bladder‐sparing outcomes in patients who are unsuitable for radical cystectomy, thereby addressing an unmet need in the management of high‐risk bladder cancer.

### 8.3 Gene Therapy

Gene therapy is a key focus in bladder cancer research, with three promising agents in advanced clinical stages.

Nadofaragene firadenovec (adstiladrin) is a gene‐based therapy using a nonreplicating adenovirus to deliver the IFN‐alpha2b gene [131]. In Study CS‐003, among 98 evaluable patients with BCG‐unresponsive NMIBC, the complete response rate was 51% (95% confidence interval: 40.7%–61.3%) at 3 months after initial treatment, with a median duration of response of 9.7 months (range: 3–52+ months), and 46% of responders remained in response for ≥ 12 months. Common adverse reactions (occurring in at least 10% of patients) included instillation site discharge, fatigue, bladder spasm, micturition urgency, hematuria, chills, pyrexia, and dysuria. Twenty percent of patients experienced grade 3 or 4 adverse reactions, with hypertension being the most common (2.5%). Treatment was discontinued in 1.9% of patients due to adverse reactions, and no patient died within 4 months of the last dose. It provides a new therapeutic option for patients with BCG‐unresponsive NMIBC with CIS who are ineligible for cystectomy [132].

Another agent, CG0070, is an oncolytic immunotherapy that has been evaluated in multiple clinical trials for BCG‐unresponsive high‐risk NMIBC. It is an Ad5‐based oncolytic vaccine engineered to express granulocyte‐macrophage colony‐stimulating factor (GM‐CSF) and replicate selectively in tumor cells with mutated or deficient retinoblastoma (Rb) protein. The E2F‐1 promoter, active only in cancer cells lacking functional Rb, controls viral replication and GM‐CSF expression, ensuring tumor selectivity [133]. Updated results from the Phase III BOND‐003 trial (NCT04452591) in BCG‐unresponsive high‐risk NMIBC with CIS (with or without papillary disease) showed a 75% CR rate at any time, with 46% maintaining CR at 12 months and durable responses observed in 52, 29, and 14 patients at 6, 12, and 21 months, respectively. The therapy was well‐tolerated, with no grade ≥ 3 treatment‐related AEs, and 95% of patients completed all protocol treatments without treatment‐related discontinuations [134].

BC‐819 (inodiftagene vixteplasmid), or DTA‐H19, is a plasmid DNA with Diphtheria Toxin A regulated by the H19 promoter combined with polyethylenimine (PEI) that selectively targets urothelial carcinoma cells through H19 promoter overexpression. The Phase II CODE trial (NCT03719300) in BCG‐unresponsive NMIBC (*n* = 32) demonstrated: (1) The complete response rate in CIS patients was measured at 12 weeks (primary endpoint) with durability assessed through 48 weeks; (2) Secondary endpoints showed high‐grade recurrence‐free rates at 12, 24, 36, 48, 72, and 96 weeks; (3) Safety evaluation over 9 months revealed adverse events graded by CTCAE v5.0, with quality of life monitored via EORTC QLQ‐C30 and NMIBC24 questionnaires. The study design (single‐arm, multicenter) achieved its primary objective, though it was terminated early, providing proof‐of‐concept for BC‐819's mechanism‐inducing tumor‐specific apoptosis while sparing normal urothelium [135].

## 9 Conclusions

Although BCG serves as the standard adjuvant therapy for intermediate to high‐risk NMIBC following TURBT, significantly improving clinical outcomes by activating the immune system and enhancing local immune‐inflammatory responses, it still has limitations [55]. Approximately 30% to 45% of patients are unresponsive or develop resistance to BCG, some high‐risk patients still experience tumor recurrence after BCG treatment, and it may cause local and systemic side effects such as bladder irritation, hematuria, and fever [10].

For BCG‐unresponsive patients, multiple FDA‐approved or guideline‐recommended treatment options are currently available. Immune checkpoint inhibitors like pembrolizumab have demonstrated high complete response rates in clinical trials, emerging as an important choice for patients unable to tolerate surgery [76]. Gene therapies such as nadofaragene firadenovec, which delivers interferon genes via adenovirus, provide a new avenue for patients unsuitable for cystectomy [132]. The IL‐15 superagonist fusion protein Anktiva, when used in combination with BCG, has achieved high complete response rates and long‐term progression‐free survival in clinical trials [94].

In the future, the treatment of BCG‐unresponsive NMIBC will focus on innovations in multiple areas. In immunotherapy, clinical trials of combination therapies such as BCG with PD‐1/PD‐L1 inhibitors are continuously advancing. In gene therapy, investigational drugs like CG0070 and BC‐819 are expected to further improve efficacy. In the field of targeted therapy, drugs targeting specific molecular targets such as FGFR inhibitors and MetAP2 inhibitors are constantly emerging, and precision treatment strategies combined with genetic testing are gradually maturing. Additionally, the development of personalized treatment plans, optimization of multidisciplinary combined treatment models, and research on novel drug delivery systems will offer more possibilities for improving patient prognosis and quality of life.

## Author Contributions

**Xiaotong Huang:** writing – original draft, writing – review and editing. **Xuan Wang:** writing – original draft, data curation. **Zihe He:** data curation. **Yishu Huang:** data curation. **Bing Hu:** data curation. **Weiying Chen:** funding acquisition, writing – review and editing. **Haifang Du:** writing – review and editing, project administration, funding acquisition, supervision.

## Ethics Statement

The authors have nothing to report.

## Conflicts of Interest

The authors declare no conflicts of interest.

## Data Availability

The data that support the findings of this study are available from the corresponding author upon reasonable request.
